# Lytics broadcasting system: A novel approach to disseminate bacteriophages for disinfection and biogenic hydrogen sulphide removal tested in synthetic sewage

**DOI:** 10.1016/j.rineng.2021.100314

**Published:** 2022-03

**Authors:** Amrita Salim, K. Sindhu Shetty, H. Febin, Nourin Sameed, Sanjay Pal, Bipin G. Nair, Ajith Madhavan

**Affiliations:** School of Biotechnology, Amrita Vishwa Vidyapeetham, Kerala, 690525, India

**Keywords:** Bacteriophage, Biogenic H_2_S, Disinfection, Lytics broadcasting system, Wastewater

## Abstract

Owing to their selective nature, bacteriophages are prospective in targeted wastewater disinfection. Other potential applications include the removal of biogenic malodour and the mitigation of corrosion in sewerage pipelines. Nevertheless, its applications are ridden with challenges, the most prominent of which is scaling up. Towards that end, effective methodologies are required for dispersing phages into wastewater. The study describes a device arbitrarily named Lytics Broadcasting System. In principle, the device contains phages that can be continuously dispersed into wastewater. The modified version is called Bacteriophage Amplification Reactor, which operates with both phages and their respective hosts, ensuring continual production and dissemination of phages. Both prototypes utilize 0.22 μm cellulose membranes as an interface through which phage diffuse passively and selectively owing to its smaller size and established through membrane-overlay method. In the study, previously reported bacteriophage φPh_Se01 and *Salmonella enterica* were used. A reduction of 3–4 log was achieved with both the prototypes after 48 h of operation in 1 L of augmented synthetic sewage. Subsequently, the biogenic H_2_S produced by *Salmonella enterica* was reduced by 64–74% indicating its utility for targeted disinfection and malodour mitigation of wastewater. This study aims to provide a framework for the development of scalable prototypes of Lytic Broadcasting Systems for real-world wastewater applications.

## Highlights

• Phages hold potential in disinfection and malodour removal in wastewater.
• Lytics Broadcasting System (LBS) passively and selectively disseminates phages.
• 0.22 μm membranes renders the LBS selective to phage dissemination.
• Bacteriophage Amplification Reactor (BAR-LBS) is a differentially operated LBS.
• 4-log and 3-log reduction of *Salmonella enterica* by LBS and BAR-LBS respectively.

## Graphical abstract

**Figure:**
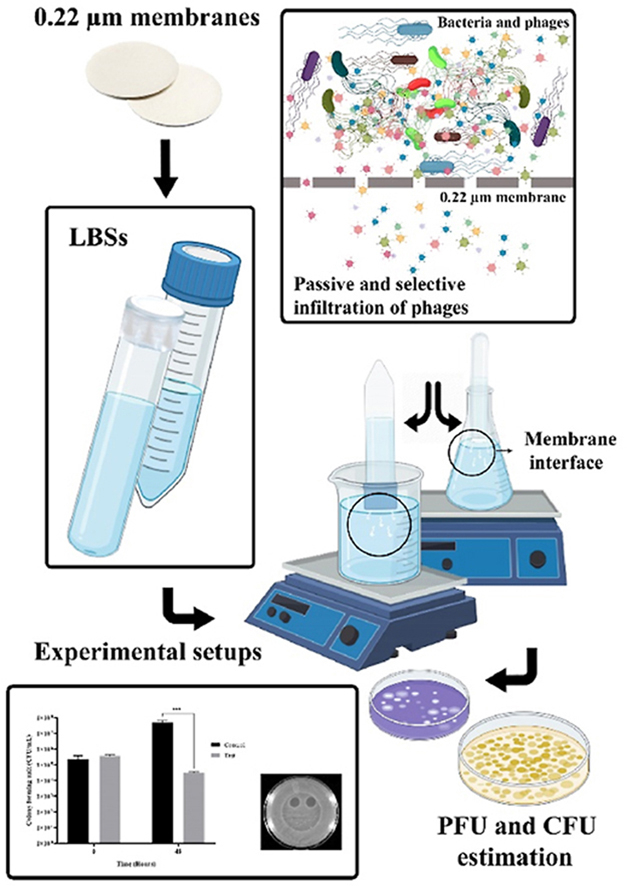

## Abbreviations

AMR: Antimicrobial resistance
BAR: Bacteriophage amplification reactor
CFU: Colony forming unit
H_2_S: Hydrogen sulphide
LBS: Lytics broadcasting system
φPh_Se01: *Salmonella* phage 01
PFU: Plaque forming unit
SRB: Sulphate reducing bacteria
WWTPs: Wastewater treatment plants

## 1 Introduction

Classical phage therapy has become a valuable alternative therapeutic tool in the growing face of antibiotic resistance with a broad range of biocontrol applications. Currently, the application of bacteriophages is primarily employed in clinical conditions such as cystic fibrosis [1,2], burn wound infection [3], impaired wound healing and infection of dental root canals [4,5]. However, there has been a recent surge in the application of phages in the field of food [[6], [7], [8]], agriculture [9,10] and aquaculture [11,12]. In recent times, the concept of the application of phages in the treatment of wastewater is gaining traction [[13], [14], [15]]. It is known that there are many chemical processes available for wastewater treatment (usage of chlorine/peracetic acid). However, residues in treated water from these compounds and their disinfection-by-products may pose a risk to humans and aquatic ecosystems [16]. Poor specificity of these chemical methods aggravates the problem further by being detrimental to the activity of other desirable bacteria involved in the natural biodegradation of wastewater. On the other hand, wastewater treatment practices that avoid or reduce the use of chemical disinfectants, such as ozone and UV irradiation, are limited by reliability, cost, and their applicability to large-scale operations [17]. Considering these challenges, the application of natural disinfection processes could represent a more viable solution to improve the specific removal of infectious bacterial pathogens from wastewater [18,19]. In this milieu, bacteriophages- the bacterial viruses with several ideal characteristics viz. specificity, the ability to kill the bacterial host rapidly, adaptability, auto-dosing, and their natural availability in any ecosystem make them a competent alternative for the disinfection of wastewater [20].

Recently, wastewater and wastewater treatment plants (WWTPs) are considered important reservoirs of bacteria harboring potentially transferable antibiotic-resistance genes into the environment [[21], [22], [23]]. Since WWTPs treat sewage from different sources, they facilitate the horizontal transfer of genes among different pathogenic bacterial species [24]. Removal of antimicrobial-resistant strains (AMR) in conjunction with effective monitoring would help in containing the spread of infection by these multidrug-resistant superbugs. Other than infection, malodour and corrosion of sewers are the major aspirational, operational, and maintenance problems with the wastewater treatment facilities. One of the major sources of foul odour is hydrogen sulfide produced in wastewater by sulphate reducing bacteria (SRBs). Selective elimination of infectious organisms and biogenic malodour mitigation could be effectively employed in wastewater treatment facilities by phages as discussed in our previous study [25]. An added advantage is that phages can minimize the selective pressure imparted by residual antibiotics to AMR bacteria and their subsequent propagation in wastewater. Nevertheless, phage-based methods should be amenable to scale-up and compatible with different settings, such as centralized and decentralized treatment systems. Central to that is their ability to disseminate phages, maintain a high titre in wastewater, and their ability to withstand harsh conditions.

Few methodologies that can be adopted for the dissemination of bacteriophages include immobilization [26,27], encapsulation [28,29], and use of liquid suspensions, for continuous production and dissemination of phage particle [19]. With phages immobilized or encapsulated, the major drawback is the complexity of the process. It requires sizeable time, potential loss of phage titre, and considerable resources to execute [27]. The application of liquid lysate, however, ensures high titre phage dispersal over a protracted period of time without the need for retrieval of immobilization matrices and/or intermittent addition of phage formulations. Nevertheless, such a direct application of phage liquid preparation would suffer from the disadvantage of being energy intensive owing to their off-site production and frequent addition. Hence it is imperative to develop an energy-neutral system for phage dissemination along with effective monitoring techniques. In an ideal system, phages would be held in a contraption that could also hold their respective hosts and such an interface could be used to transmit them selectively without the use of energy. Bacteriological membrane filters can effectively serve as an interface for selective diffusion of phages while simultaneously preventing infiltration of the bacterial hosts.

This study shows that 0.22 μm cellulose membranes have the potential for passive diffusion of phages. Phage particles are selectively and passively dispersed into synthetic sewage due to the limiting pore size and lack of pressure. The experimental prototypes used in the study were called Lytics Broadcasting System (LBS). Another prototype, the Bacteriophage Amplification Reactor (BAR-LBS), holds both phages and their host cells, thus serving as a generator of the lytic agents (phages) for continuous dispersal of phages into the simulated sewage through a 0.22 μm membrane that acts as an interface by which passive diffusion of the phages occurs due to their submicroscopic size. Two prototypes were built to evaluate the potential for field applications.

## 2 Materials and methods

### 2.1 Bacterial strain and bacteriophage

Multi-drug resistant clinical strain of *Salmonella enterica* was used in the study. The strain was cultured in the Luria Bertani (LB) broth at 37 °C for 16–18 h in a shaking incubator at 250 rpm. The culture was subcultured in *Salmonella*-*Shigella* Agar (SS Agar, HiMedia, India) and stored at 4 °C throughout the experiment. Bacteriophage φPh_Se01 previously reported for its ability to reduce biogenic H_2_S produced by *S. enterica* was employed for the disinfection experiments [25]. The phage was stored and maintained at 4 °C for immediate use and at −80 °C for long term storage.

### 2.2 Membrane overlay to determine passive diffusion of phage

**Fig. 1:**
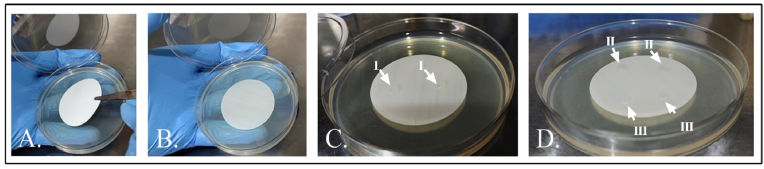
A. & B. Membrane overlay on LB agar to determine phage/bacterial infiltration across the membrane. C. Bacteria (I). D. Phage (II) and SM buffer (III).

Using 0.22 μm cellulose membrane filters (47 mm, diameter, Millipore, India) the passive diffusion of phage φPh_Se01 was qualitatively analyzed by the bacterial lysis experiment using the membrane overlay method as previously reported with modifications [30,31]. Initially, the LB agar plates were flooded with the host bacteria and the plates were incubated for 2 h at 37 °C for drying the culture. Briefly, the membrane was placed on the surface of the pre-incubated host bacterial lawn, and 20 μL of phage lysate (φPh_Se01) was dropped over the membrane (Fig. 1D). The SM buffer was used as a negative control, and the plates were incubated for 5–6 h at 37 °C. Following incubation, the membrane was removed from the plate, and the incubation was further carried out overnight at 37 °C to check for the presence of bacterial lysis by the infiltrating phages through the membrane. Subsequently, we also tested the possible permeability of bacteria through the membrane. Briefly, a membrane was placed on the surface of a plane LB agar plate without any bacterial lawn underneath and the liquid culture of 20 μL was dropped on the surface of the membrane and left undisturbed to dry (Fig. 1C). The plates were then incubated at 37 °C for 24 h and following incubation, the membrane was lifted vertically to determine the growth of bacterial colonies on the agar surface.

### 2.3 Design of laboratory scale membrane-based lytic broadcasting system (LBS)

**Fig. 2:**
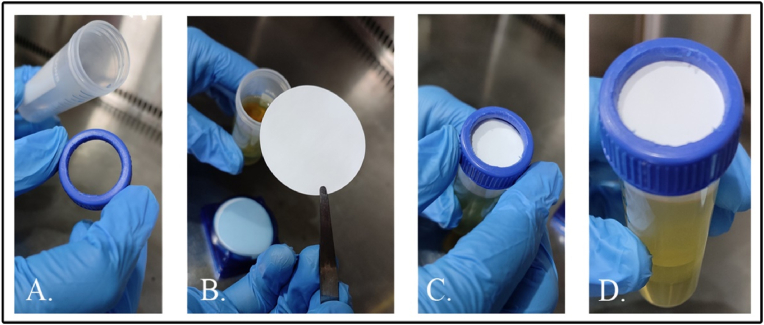
Laboratory scale LBS assembly using a 50 mL conical centrifuge tube and 0.22 μm cellulose membrane filter. A. Modified lid, B. 0.22 μm cellulose membrane filter, C. Mounting of the membrane, D. Assembled LBS prototype.

A 0.22 μm membrane-based lytic broadcasting system was developed to disseminate phages for the potential application of disinfection of enteric bacteria in wastewater. The first set-up includes a 50 mL sterile conical centrifugation tube to hold phage lysate or its respective bacterial host. To secure the 0.22 μm membrane onto the mouth of the tube, a wide perforation was cut out on the screw cap, and the membrane was mounted and held in place by tightly securing with its cap (Fig. 2). Aseptically assembled LBS was suspended in such a way that the membrane is in contact with the surface of the media (buffer/synthetic sewage) to facilitate the diffusion of phages. A modified LBS with membrane secured onto the boiling tube was used in large-scale experiments (1 L) to monitor the production of H_2_S since it can be accommodated in a conical flask and its mouth hermetically sealed (Fig. 4).

### 2.4 Determination of phage/bacterial infiltration rate

**Fig. 3:**
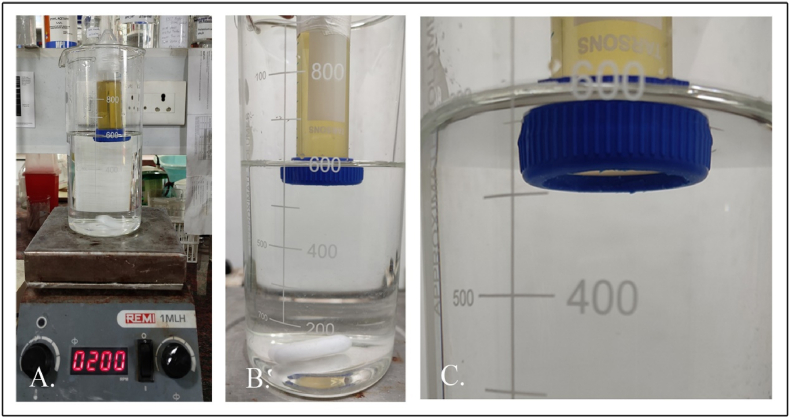
Experimental set-up designed to determine the infiltration rate of phage/bacteria using an LBS. A. LBS suspended in 600 mL SM buffer/0.85% saline, B and C fluid-membrane interface.

A study of the rate of phage/bacterial infiltration through an LBS was conducted for 24 h. The experiment was such that an LBS was initially charged with 40 mL phage lysate of φPh_Se01 with an average titre of 3.9 × 10^11^ PFU/mL in a conical centrifugation tube. LBS was further introduced into a 600 mL SM buffer (phage buffer, pH 7.4) containing beaker without completely immersing the membrane-mounted tube as mentioned in section 2.3. The buffer was mixed uniformly using a magnetic stirrer at ∼250 rpm in an aseptic condition and the sample of 1 mL was retrieved at a regular interval of 1 h for a period of 6 h and then at 24 h. The sample at each time point was diluted in the SM buffer and the dilutions were then used to determine the phage titre against the bacterial host. The plates on incubation at 37 °C were checked to calculate the average rate of phages passively filtrated through the membrane-bound LBS over a period of 24 h (Fig. 3). Similarly, the bacterial infiltration rate was determined in the same manner. Briefly, an LBS with only the bacterial culture of 40 mL with an OD_600nm_ 1.0 (∼1 × 10^9^ CFU/mL) was introduced into a 1 L beaker with 600 mL of 0.85% saline maintained in a sterile environment. The setup was continuously mixed as mentioned earlier and the sample of 1 mL was taken for the same amount of time as that of the phage infiltration experiment. The collected sample was diluted in 0.85% saline and the dilutions were plated in SS agar to determine the rate of infiltration of bacteria through the membrane of the LBS. Subsequently, for rapid detection, the sample was also subjected to a resazurin-based fluorometric assay to check for the presence of the infiltrated bacteria, if any.

### 2.5 Disinfection and biogenic hydrogen sulphide reduction using LBS in synthetic sewage

**Fig. 4:**
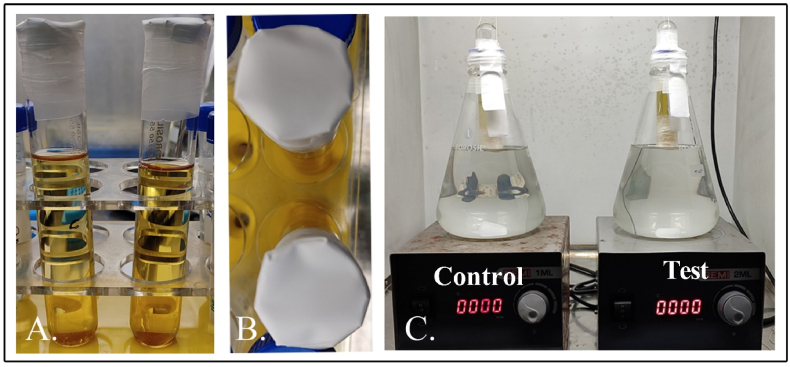
Pilot scale LBS (A) and (B). C. 1 L scale treatment of augmented synthetic sewage.

Tests were conducted with 1 L of simulated synthetic sewage (peptone, 160 mg; meat extract, 110 mg; urea, 30 mg; anhydrous dipotassium hydrogen phosphate (K_2_HPO_4_), 28 mg; sodium chloride (NaCl), 7 mg; calcium chloride dihydrate (CaCl_2_·2H_2_O), 4 mg; magnesium sulphate heptahydrate (MgSO_4_·7H_2_0), 2 mg; L-Cysteine, 10 mM) [32] augmented with 5 mL *Salmonella* culture (OD_600nm_ 2.3) to determine whether LBS effectively disinfected and reduced biogenic hydrogen sulfide. In brief, LBS was prepared in a sterile 55 mL boiling tube comprising 40 mL of phage lysate with a titre of 5.96 × 10^9^ PFU/mL, while phage broth served as a negative control. We then affixed sterile 0.22 μm membrane filters onto the mouth of the tubes by using UV sterilized parafilm as mentioned in 2.3. Following this, the tubes were inserted into flasks containing *Salmonella* (OD_600nm_ 2.3) simulated synthetic sewage, such that the tube's brim touched the synthetic sewage without being completely immersed as shown in Fig. 4C. The sample was mixed using magnetic beads at ∼200 rpm and the flasks were sealed with parafilm to ensure adequate H_2_S production. Samples were collected beforehand from both control and test sets in order to determine the number of bacteria at 0 h. The experimental setups were incubated at room temperature for a period of 48 h. Following the incubation, a 5% lead acetate strip (23 × 4 cm) was introduced into the flasks for a period of 2 h to determine the production of H_2_S in both setups; the difference in the integrated density was quantified using Image J as described in the previous studies [25]. Subsequently, 1 mL of the sample was also collected from each set up to determine the bacterial cell viability count and phage titre. The phage-treated sample and untreated control were also monitored for chemical characteristics including biological oxygen demand (BOD), chemical oxygen demand (COD), total dissolved solids (TDS), total suspended solids (TSS), volatile organic content (VOC), and pH.

### 2.6 LBS as a bacteriophage amplification reactor (BAR-LBS)

Bacteriophage amplification reactor was developed within an LBS by feeding the system with 40 mL mixture containing the phage and its respective host in this case, *Salmonella enterica* (1.1 × 10^8^ CFU/mL). The phage φPh_Se01 with a titre of 4.3 × 10^8^ PFU/mL was charged in the test BAR-LBS system. The control consisted of an LBS charged only with the bacterial host supplemented with phage broth. As mentioned in section 2.5, LBS was developed in a boiling glass tube and was later introduced into a 1 L synthetic sewage augmented with the host (OD_600nm_ 1.1). The sample was mixed to avoid sampling errors and the flasks were made airtight using parafilm to ensure H_2_S production. The samples (1 mL) were taken at 0 h and 48 h to be plated on SS agar from both control and test and the corresponding titre of phage in the test was also determined. Subsequently, 5% lead acetate strips were inserted for 2 h after 48 h of treatment, and the concentration of lead sulphide formed on exposure to the H_2_S produced was quantified in terms of integrated density using ImageJ as mentioned above.

### 2.7 Statistical analysis

All the experiments were analyzed using GraphPad Prism Version 8.0.2. The level of significance was defined at *p* ≤ 0.05. All data sets are presented as a mean value ± standard deviation (SD).

## 3 Results and discussion

### 3.1 Passive infiltration of phage φPh_Se01

**Fig. 5:**
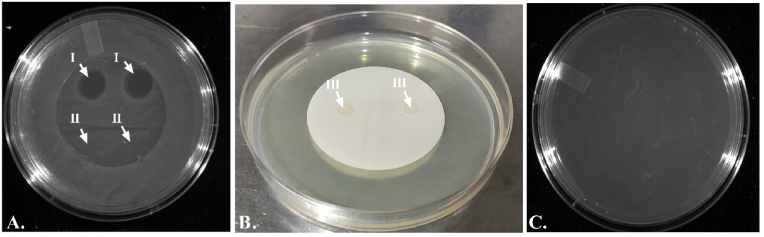
Membrane overlay method for Passive diffusion of phage/bacteria through 0.22 μm membrane. A. The plate shows the zone of lysis indicating passive infiltration of phages through the membrane (I), while the control SM buffer did not show any indication of passage through the membrane (II). B. The image shows the growth of retained bacteria over the membrane on incubation at 37 °C for 24 h. (III). C. The absence of bacterial growth after removing the membrane indicates selective permeability of only phages across the membrane.

The membrane (0.22 μm) could efficiently diffuse the phages passively dropped over the surface of the membrane filter (Fig. 5A). This was observed as a clear zone of lysis over the bacterial lawn on removing the membrane while the control SM buffer did not show any effect on the bacterial growth (Fig. 5A). The selectivity of the membrane was tested using bacteria where the liquid bacterial culture failed to diffuse through the membrane showing no growth in the LB agar after incubation for 24 h, rather the growth was observed on the surface of the membrane (Fig. 5B, C). The selectivity of 0.22 μm membrane to phages could be ascribed to the narrow pore size of the membrane as well as to the much smaller size of the phages (0.02–0.07 μm) compared to the larger bacterial cells (0.5–2 μm) [33]. Passive diffusion in the absence of pressure could serve as an ideal characteristic in the development of an energy-efficient lytics broadcasting system. Conventional wisdom indicates that pressure above 1 bar is required to extrude the contents across the membrane [34]. However, the current study reiterates the slow, effortless, and passive diffusion of submicroscopic particles including viruses like phages.

### 3.2 Phage/bacterial infiltration rate

**Fig. 6:**
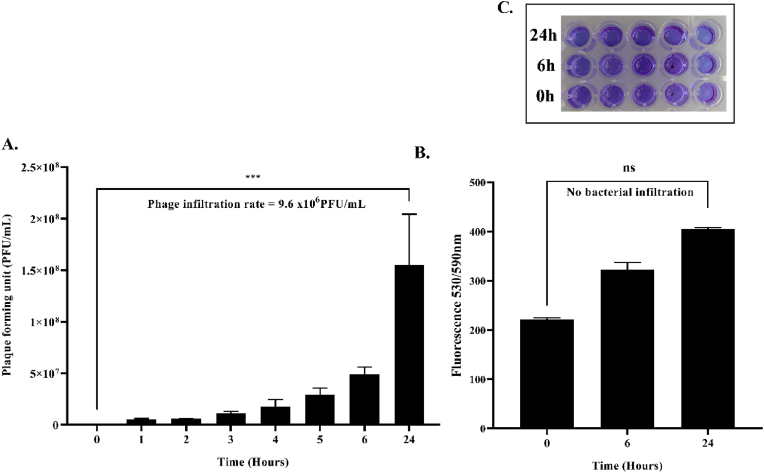
A. Passive infiltration rate of phages over a period of 24 h through LBS (p < 0.001). B. Absence of infiltration of bacteria through LBS is determined by resazurin assay (p > 0.05). C. The 96-well plate shows the absence of bacterial viable activity by resazurin indicating the absence of infiltration of bacteria from the LBS membrane into 0.85% NaCl.

The rate of infiltration of phages through 0.22 μm membrane was tested using an LBS where the phages could passively diffuse through the membrane into the surrounding medium (SM buffer), and the average titre diffused per hour was determined to be 9.3 × 10^6^ PFU/mL (Fig. 6A, p < 0.001). We also showed the inability of the bacteria to infiltrate through the membrane, as there was no bacterial growth in the plate assay as well as in the resazurin assay even after 24 h, ensuring successful filtration of the host bacteria by the membrane (Fig. 6B). This experiment corroborates our earlier experiment on the selectivity of 0.22 μm membrane in the infiltration of viral lysate that could be potentially utilized as an energy-efficient dissemination strategy for the specific disinfection of wastewater. This particular model is least affected by membrane biofouling issues, as phages have already been reported to act as an antimicrobial agent against membrane biofouling in ultrafiltration units [31,35,36].

### 3.3 Disinfection of synthetic sewage by LBS/BAR-LBS

**Fig. 7:**
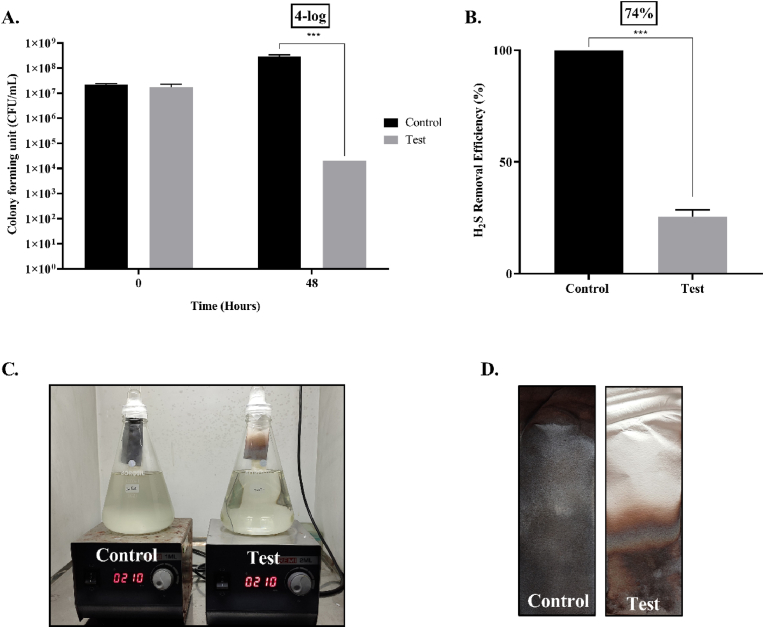
A. The reduction in CFU by 4-log after the introduction of LBS into the augmented synthetic sewage after 48 h (p < 0.001). B. Reduction in production of H_2_S in test sample by 74% after 48 h, compared to the control set up (p < 0.001). C. Photographic images show a clear difference in turbidity as well as lead sulphide deposition on the lead acetate strip, an indication of reduction in the H_2_S production between the control (left) and the test (right) setup of an LBS. D. The lead acetate strip showing a reduced lead sulphide precipitation between the phage treated sample (right) and the untreated control (left) after 48 h using an LBS.

LBS's potential for disinfecting bacteria of interest in 1 L synthetic sewage indicates its utility in eliminating antimicrobial-resistant and infectious bacteria like *Salmonella enterica*, which also produces biogenic H_2_S that may contribute to malodourous wastewater. The system was efficient in reducing the bacterial count by 4-log in 48 h when compared with the control where the LBS was charged only with phage broth (Fig. 7A, p < 0.001). We observed a high phage titre (10^9^ PFU/mL) in the test in 48 h, indicating a gradual diffusion of phages from the LBS and subsequent amplification in the augmented synthetic sewage resulting in the lysis of the bacterial host. We also tested the reduction in H_2_S production using lead acetate strip test showing 74% reduction after 48 h (Fig. 7B, p < 0.001), deduced from the integrated density differences in the lead acetate strips, between the phage treated synthetic sewage and the corresponding control lacking phages in the LBS for appropriate disinfection and H_2_S control (Fig. 7C). The control set-up had an expected decrease in parameters, viz. BOD, COD, total suspended solids, and total dissolved solids as given in Table 1. As opposed to the parameter control mentioned above, the test setup revealed an increase. In both setups, volatile organic compounds and pH were the same, however. This outcome is consistent with the observation that, in the control set-up, the target organism's load remained higher due to the absence of lysis by bacteriophages. Consequently, the parameters are reduced since these organisms consume nutrients/organic matter. However, in the test set-up, the infiltrated phages upon lysing the bacterial cell (4-log reduction) resulted in reduced bioremediation and an increase in the organic content, as reflected in the above-mentioned chemical parameters.

**Table 1:** Chemical characteristics of synthetic sewage from both control and test setups of LBS after 48 h of treatment.

Sl. No.	Chemical Parameters	Control Setup (mg/L)	Test Setup (mg/L)
1.	TSS	62	110
2.	TDS	1225	1595
3.	BOD	762	848
4.	COD	1569	1670
5.	VOC	<0.005	<0.005
6.	pH	7.0	7.0

The system can be further improved by employing a cocktail of one or more phages that can undoubtedly impact the performance of the LBS positively while translating into real wastewater treatment. The scope of the study involves targeting specific pathogens that can contribute to malodour and to an extent infection to both humans and animals, especially the enteric bacteria. Moreover, such a system can make the process of application of phages easier and far more user-friendly for the workers in the wastewater treatment facilities, unlike other chemical methods [37,38]. Furthermore, membrane-based LBS can be scaled to treat larger volumes of sewage, while technologies like ozone and UV-irradiation are restricted by their applicability in treating higher volumes of wastewater [17].

**Fig. 8:**
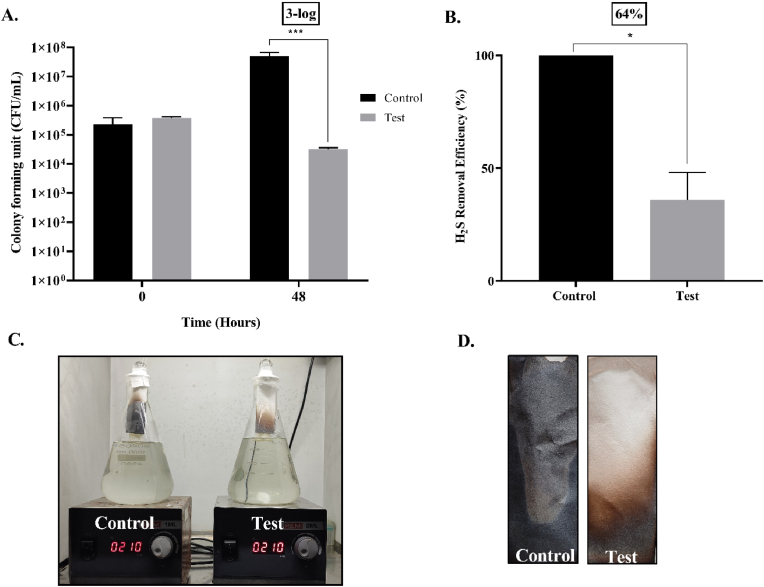
An LBS as a bacteriophage amplification reactor (BAR-LBS) A. 3–log reduction in the viable count of *Salmonella* after 48 h of treatment in BAR-LBS where both host and the bacteriophages were added (p < 0.001). B. The reduction in biogenic H_2_S after phage treatment with a BAR-LBS was evaluated by a lead acetate strip that showed 64% decrease in the biogenic H_2_S after 48 h in the phage-treated experimental setup (p < 0.01). C. The turbidity of control (left) and test (right) after 48 h of phage treatment differed significantly. D. The difference in lead sulphide formation is due to reduced H_2_S in the test (right) compared to the control (left).

**Fig. 9:**
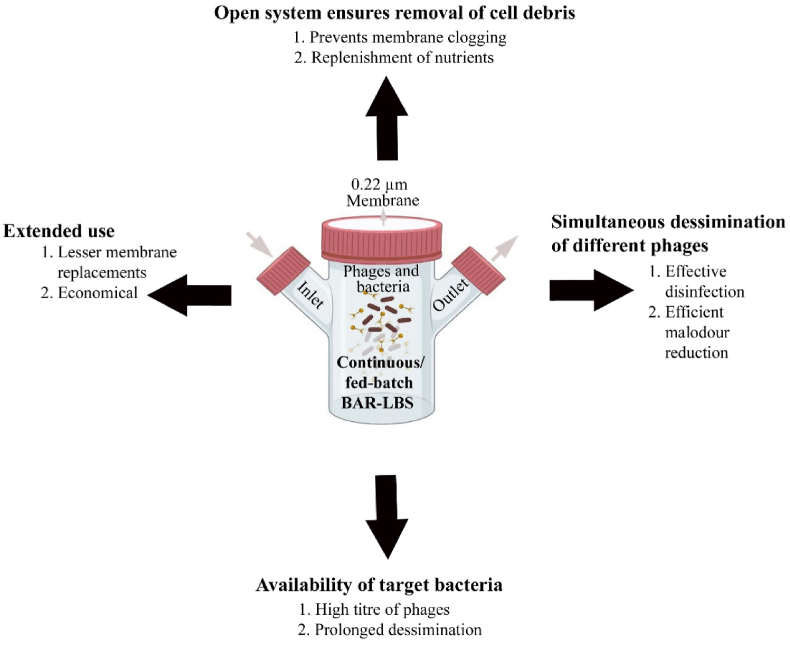
Schematic representation of continuous/fed-batch BAR-LBS for prospective field application in decentralized and centralized wastewater treatment facilities.

BAR-LBS is a variant of LBS that can guarantee continuous amplification of bacteriophages within the LBS in the presence of its respective host, and so maintain its efficiency without exhausting its capacity as the phages deplete over time. In this model, we achieved approximately a 3-log reduction in the treatment, while the control was affluent with bacteria in the absence of phages (Fig. 8A, p < 0.001). The presence of the phages (4.5 × 10^7^ PFU/mL) after 48 h in the test setup indicates their diffusion through the membrane. The reduced biogenic H_2_S (∼64%) yield after 48 h (Fig. 8B, p < 0.01) in the treatment setup indicates the potential of the system not only in disinfection but also in the biocontrol of biogenic H_2_S in field trials for biological wastewater treatment. Nevertheless, the 1-log difference between phage-alone LBS and BAR-LBS may be attributed to the accumulation of cell debris on the membrane of the BAR-LBS from lysed bacteria. Thus, the infiltration efficiency of phages can be affected as is evident in the overall difference in bacterial load, phage titre, and subsequent H_2_S reduction in comparison with the phage-alone LBS. It may be possible in the future to resolve this issue by making the model a continuously running system in which hosts bacteria are enriched and mixed simultaneously with their contents (Fig. 9). This prevents the debris from settling, while at the same time ensuring multiplication of phages more efficiently and infiltration more effectively. We also expect lesser membrane fouling because of continuous degradation of the cell wall debris/cellular biofilm by the hydrolases coming out of host cells, unlike the usual membrane bioreactor where non-target live bacteria make biofilm on the membrane and clog the membranes. As a consequence, the phages diffuse passively into the surrounding environment, maintaining a high m.o.i (multiplicity of infection, i.e., ratio of phages to the host) against the targeted bacteria. The system also provides flexibility for simultaneous dissemination of cocktails of phages that could address issues of bacterial resistance, incomplete disinfection and malodour reduction. Continuous systems can also influence economics positively through extended usage of the device and minimal membrane replacements. Furthermore, such a system of targeted biocontrol by phage can be applied to anaerobic digesters to combat sulphur reducing bacteria (SRBs) that otherwise compete with methanogens, reducing methane production as well as increasing corrosion and odour problems [39]. This proposition is a cost-effective strategy for enhanced resource recovery and longevity of infrastructure [40,41]. Additionally, the use of lytic phages in wastewater treatment facilities can effectively stabilize the biomass bulking and foaming caused by filamentous bulking bacteria like *Gordonia* spp., *Nocardia* spp. [14,15], and *Haliscomenobacter* spp. [13] as demonstrated in several other studies.

## 4 Conclusion

Membrane-based LBSs were developed as devices for the translation of phage-based solutions in the treatment of pathogenic bacteria in wastewater through a targeted approach. The device would help in addressing problems of infection and malodour associated with wastewater treatment, especially at the domestic level. A notable advantage of these systems is operational flexibility, such as BAR-LBS, which enables phages and their respective hosts to be loaded into an LBS, which ensures continuous and specific release of phages. LBS and BAR-LBS reduced *Salmonella enterica* by 4-log and 3-log, respectively, in a 1 L of synthetic sewage after 48 h, with subsequent reductions of 64–74% in biogenic H_2_S for both prototypes.

The most essential component of the prototypes is a 0.22 μm membrane. The selective and passive nature of phage infiltration across membranes was well established before the development of LBS and BAR-LBS. Bacteriophages were shown to pass through a membrane passively because of their smaller dimensions compared to their bacterial targets and provided effective bacterial clearance when continuously operated for 48 h. Apart from the utility, the laboratory-scale prototypes provided sufficient insight for the expansion of these models for application in real wastewater settings. Because of their modular nature, LBSs (or BAR-LBS) can be utilized to disseminate unitary or cocktails of phages to domestic wastewater systems in a cost-efficient manner. Moreover, an LBS's energy-efficient mode of operation allows it to be more scalable than other bacteriophage-based technologies like immobilization, encapsulation, or high-pressure ultrafiltration by energy-intensive pumping. However, further research needs to be carried out to validate the performance of the continuous system for large-scale wastewater disinfection in order to establish this as a technology to be adopted in wastewater treatment facilities.

## Credit author statement

**Amrita Salim**: Conceptualization, Methodology, Investigation, Validation, Data Curation, Formal Analysis, Visualization, Writing-Original Draft Preparation, Writing- Review and Editing. **Sindhu Shetty K**: Conceptualization, Methodology, Writing- Review, and Editing. **Febin H**: Methodology, Validation. **Nourin Sameed**: Validation. **Sanjay Pal**: Conceptualization, Writing – Review, and Editing, Project Administration, Funding Acquisition. **Bipin G. Nair**: Funding acquisition, Project Management, and Administration. **Ajith Madhavan**: Conceptualization, Methodology, Visualization, Supervision, Writing-Original Draft Preparation, Writing- Review and Editing.

## Declaration of competing interest

The authors declare that they have no known competing financial interests or personal relationships that could have appeared to influence the work reported in this paper.
